# Effectiveness of continuous and pulse mode of ultrasound therapy in temporomandibular disorders associated myalgia—a randomized controlled study

**DOI:** 10.22514/jofph.2025.007

**Published:** 2025-03-12

**Authors:** Bhawna Saini, Ambika Gupta, Harneet Singh, Suman Bisla, Suriya Nagarajan, Komal Kumia

**Affiliations:** ^1^Department of Oral Medicine and Radiology, Faculty of Dental Science SGT University Budhera, 122505 Gurugram, India; ^2^Department of Oral Medicine and Radiology, Post Graduate Institute of Dental Sciences, 124001 Rohtak, India

**Keywords:** Myofascial pain, Ultrasonic therapy, Temporomandibular joint disorders, Orofacial pain

## Abstract

**Background**: Temporomandibular disorders associated myalgia (TMD-M) is one of the most common patient complaints in clinics. Because of the disease’s multifactorial etiology and complexity,extensive understanding is required to determine an appropriate treatment protocol. **Methods**: The current randomized comparison study included 80 patients who presented to the outpatient department with a TMD-M complaint. Patients were randomly assigned to one of two groups: continuous therapeutic ultrasound or pulsed therapeutic ultrasound, according to a standard protocol. The key outcome measures were pain intensity (visual analog scale (VAS), 0–10 cm) and muscle pressure pain threshold (PPT). Secondary outcome assessments included changes in maximal mouth opening, functional movements, and depression (Beck Depression Inventory (BDI)). A descriptive analysis was performed on the dataset to get data estimates for all variables. **Results**: The means of the differences in the two group’s values were compared. Intergroup comparisons for normally distributed data were performed using independent sample *t*-tests, and intragroup comparisons using repeated-measures Analysis of variance (ANOVA). For non-normally distributed data, such as pressure pain sensitivity (PPT), BDI, left laterotrusive movement (LLT), and protrusive movement (PM), intergroup comparisons were performed using the Mann-Whitney test, and intragroup comparisons using the Friedman test followed by the Wilcoxon signed-rank test. Although the intragroup changes in visual analogue scale (VAS) score, PPT, BDI, LLT and PM were highly significant in both groups (*p * < 0.001), there was no significant intergroup difference in pain reduction, PPT, BDI, LLT or PM (*p *> 0.05). There were no significant intergroup or intragroup differences in mouth opening or right lateral movement. **Conclusions**: Both the pulse and continuous modes of therapeutic ultrasound (US) are equally effective in relieving pain. US therapy in both modes is a potent and independent therapeutic modality for the treatment of TMD-M. **Clinical Trial Registration**: NCT05211245.

## 1. Introduction

TMD is the condition of the orofacial region affecting the masticatory muscles, Temporomandibular joint (TMJ) and associated structures or both. The prevalence of TMD may vary from 10% to 72% globally, with women and students experiencing the symptoms more often than men. TMD presents clinically with a constellation of symptoms such as pain, joint sounds and impaired functional movements. Masticatory myalgia alone accounts for 25% of all cases of TMD [1].

Myalgia is considered a generalized disorder of muscle pain, and local myalgia and myofascial pain are considered subtypes [2]. It is characterized by pain and dysfunction that occur due to pathologic and functional processes within the masticatory muscles. While several mechanisms, such as unsatisfactory occlusion, missing dentition, micro and macro trauma and stress, contribute to myalgia, there is little evidence regarding gross pathophysiological changes within the muscular tissue. In approximately 25% of patients with TMDs, masticatory myalgia is the primary source of pain. Besides being the primary source of pain, myalgia may also occur secondary to TMJ pain due to protective co-contraction, vicious cycle theory, and integrated pain adaption model [1].

Due to the disease’s complex nature, understanding its pathophysiology is still challenging. A multimodal approach to reduce pain, resolve muscle spasms, and improve restricted range of motion, which includes patient education, self-care, physical therapy, intraoral appliance therapy, pharmacotherapy, behavioral and relaxation therapy, is advocated [3, 4].

The latest Diagnostic Criteria for TMD (DC/TMD) further classify myalgia as local myalgia, myofascial pain or myofascial pain with referral, differentiated by provocation testing with palpation. Local myalgia is localized only to the site of palpation. The term “Myofascial pain” is used when the myalgia spreads beyond the site of palpation but within the boundaries of the muscle. Myofascial pain with referral is the pain referred beyond the boundary of the palpated muscle [2, 3].

Therapeutic ultrasound has a frequency ranging from 750,000 to 3,300,000 Hz (0.75 to 3.3 MHz). Based on its therapeutic effects, ultrasound therapy can be categorized as thermal or mechanical. The thermal effect, which is a result of the continuous mode of US therapy, causes a transient increase in the flexibility of collagenous structures, including ligaments, tendons and joint capsules, thus leading to a decrease in pain and muscle spasm, stiffness of the joint and a temporary increase in blood flow. The pulsed mode of US results in a nonthermal effect, *i.e.*, micro-massage, which leads to segmental analgesia due to decreased central and peripheral sensitization [5]. The nonthermal effect of US can be explained by the frequency resonance theory, which states that the proteins in these structures absorb mechanical energy, thus altering their structure and function and ultimately resulting in the stimulation of phagocytosis, an increased number of free radicals, increased cell membrane permeability, cellular proliferation and the acceleration of fibrinolysis [6].

Therapeutic ultrasound (US) is a commonly used noninvasive physical therapy for treating TMD-associated myalgia. However, the scientific literature still lacks the guidelines for the use of therapeutic US for TMD-M. The US employs inaudible high-frequency mechanical vibrations converted into acoustic energy [7]. US therapy alters nerve conduction velocity and cell membrane permeability and increases the rate of tissue repair and wound healing. This leads to thermal and biophysical effects such as increased tissue extensibility, reduced calcium deposits, pain, and muscle spasms [8]. US frequency, intensity, effective radiating area of transducer head, duration of treatment, and tissue composition may influence the US dose delivered to the target tissue [9].

The therapeutic frequency range of US is 0.75–3 MHz, and most machines are generally set at 1 or 3 MHz. The low frequency (1 MHz) beam has a greater penetration depth (3–5 cm) but provides a less focused beam and is therefore used for deeper tissues or in patients with more subcutaneous fat. On the other hand, a frequency of 3 MHz is recommended for more superficial tissues at depths of l–2 cm [9]. As the masseter and temporalis muscles are located superficially, it was decided to use US waves at 3 MHz frequency for treating TMD-M. The thermal effect produced by the continuous mode causes a transient increase in the flexibility of collagenous structures, thereby leading to a short-term decrease in pain and muscle spasm, joint stiffness, and a temporary increase in blood flow. The mechanical effect produced by the pulsed mode causes micro-massage that leads to segmental analgesia due to decreased central and peripheral sensitization [5]. The ability of the US to produce long-term effects may be attributed to its central effect mediated through a reduction in nitric oxide synthase-like neurons in the nucleus propria and ventral horn of the spinal cord.

Although few studies acknowledge the use of US therapy in neck muscles are available, there is a paucity of literature on the use of therapeutic US for treating TMD-M. A systematic review and meta-analysis performed by Peng Xia *et al*. [10] (2017) revealed that continuous US was effective in relieving pain in the Myofascial pain syndrome (MPS) of the neck but was not effective in improving the range of movement. Ahmed S *et al*. [11] (2021), in a systematic review, concluded that therapeutic US had significant positive results in reducing TMD symptomology compared to Transcutaneous Electrical Nerve Stimulation (TENS) therapy. Another systematic review by Ansari S *et al*. [12] (2022) compared the efficacy of Low-level LASER therapy, TENS and therapeutic US for pain management and mouth opening (MO) in TMDs. They concluded that both TENS and the therapeutic US are effective in reducing the TMD symptoms [12]. However, better results were observed with Low-level LASER therapy than with TENS or therapeutic US.

Ilter L *et al*. [13] studied that continuous ultrasound therapy was more efficient in reducing pain at rest for myofascial pain syndrome patients as compared to sham or pulsed ultrasound therapy. Still, there is a paucity of literature evaluating therapeutic ultrasound for the management of TMD-M. Although the previous literature has established the therapeutic role of US for the management of myofascial pain compared to sham US and other physical therapies such as TENS, LASER and medication [8, 13, 14], there is a lack of concrete evidence regarding the utilization of standardized diagnostic criteria for myalgia, the duration of application of US therapy with adequate sample size and stratification. Due to the lack of large-scale randomized control trials, heterogeneity, and low-quality studies, the effect of US on TMD-M is still controversial. Moreover, it is difficult to infer the results of studies performed in other muscles. Most of the studies available in the literature have compared the effect of the US with other conventional therapies in the masseteric region and have shown good therapeutic effects as a monotherapy and an adjunct therapy [11, 12].

The study’s primary aim was to compare the effectiveness of continuous versus pulsed ultrasound in reducing the pain intensity and pressure pain sensitivity in the masseter and the temporalis muscle of participants diagnosed with TMD-M. As a secondary objective, we compared the effectiveness of continuous and pulsed US on improving mouth opening, functional capacity, and level of depression in participants with TMD-M.

## 2. Materials and methods

### 2.1 Study design

This hospital-based randomized double-blind parallel-group clinical trial was carried out in the Department of Oral Medicine. All patients who reported to the outpatient department between August 2021 and October 2022 were screened for TMD-M. 80 patients of either sex, aged 18–60 years, who fulfilled the inclusion and exclusion criteria were recruited for the study. The Institutional Ethical Committee approved the study (PGIDS/BHRC/21/27), and the trial was prospectively registered (Clinical Trials gov: NCT05211245 https://clinicaltrials.gov/study/NCT05211245?term=NCT05211245&rank=1). All the participants provided a written informed consent to participate in the study.

### 2.2 Study population

The sample size was calculated at 0.80 power and alpha error probability of 0.05 using the two-tailed test and the following formula:

$$N=\;2\;\times \;{\left[Z(1-\alpha /2)\;+\;Z(1-\beta )\;\right]}^{2}{\left(\sigma \right)}^{2}/{\left(\mu_{1}-\mu_{2}\right)}^{2}$$

A total sample size of 70 was calculated by using mean and standard deviation (SD) values ascertained from previous studies and found to be sufficient to detect a decrease in visual analog scale (VAS) pain score and an effect size of 0.6 [13]. To compensate for attrition, the sample size was increased to 80 with an allocation ratio of 1, making it 40 participants in each group.

The participants were randomly allocated into two groups using simple random sampling. A random Lottery method was used where each participant was asked to pull a chit, sealed in an opaque envelope from a box containing an equal number of both chits. The chit was marked as “A” or “B” indicating the treatment groups. Based on the chit they pulled out, the patient was allotted to either group A or B. HS was involved in the process of randomization; two investigators, BS and SN, enrolled the participants and provided treatment. Two outcome evaluators, AG and KK, who were blinded to the allocation process, were trained to standardize the evaluation technique of pain intensity, PPT, LLT, PM and Beck depression score. So, both the participants and the outcome assessors were blinded for the treatment administered in the study groups.

### 2.3 Inclusion criteria

Participants diagnosed with temporomandibular disorder myalgia of the masticatory muscles according to the DC/TMD criteria and who provided written informed consent to participate in the study were included. DC/TMD criteria describe myalgia as a pain of muscle origin that is affected by jaw movement, function, or parafunction, and replication of this pain occurs with provocation testing of masticatory muscles [2].

### 2.4 Exclusion criteria

Patients with epilepsy/seizures, radiographic changes suggestive of pathological conditions of the temporomandibular joint (TMJ), undiagnosed orofacial pain, history of treatment of TMD in past 3 months to preclude any benefit obtained from previous treatment, any type of skin lesion at the site of electrode placement, areas of impaired circulation, ischemia, areas with sensory deficits, sites of active infection or Beck Depression Score >25 were excluded from the study.

### 2.5 Study protocol

Participants were equally allocated into two groups using a simple randomization method. Both the participants and the outcome assessor were blinded to the treatment group. Patients in Group A (n = 40) received conventional continuous ultrasound using Bio-Med Inc. equipment (Bio-Med International Pvt. Ltd., Physiotherapy equipment, New Delhi, India) with a US probe 3 cm in diameter. US therapy was provided by the same investigator at 3 MHz frequency, 2.0 Watt/cm^2^ intensity for 5 minutes at each session. The probe was moved circularly over the entire muscle. Group B (n = 40) was also treated similarly to group A at 3 MHz frequency, 1.1 Watt/cm^2^ intensity, and pulsed ultrasound (at a 1:1 ratio). Both treatment groups were treated six days per week for two weeks or until the patient reported a VAS score ≤3, whichever occurred earlier (Fig. 1). VAS score ≤3 is considered mild pain and is usually managed with alternative therapies at home [15]. So, this value was accepted as the endpoint for treatment effectiveness. Masseter and temporalis muscles were considered for the measurement of outcome parameters. When the participants had myalgia in multiple maxillofacial muscles, the site of maximum pain was considered for the evaluation of the outcome parameters. However, the same treatment was administered to all the involved sites, including the patient’s neck muscles.

**Fig. 1.** Flowchart of the study protocol. VAS: visual analog scale; TMD: Temporomandibular disorders.

Participants were advised not to take any analgesics during the study period unless the pain was unbearable. The number of pain medications consumed by the patient was, recorded at the follow-up visit. No other treatment or intervention was provided during the study period to avoid any bias in the outcome.

The primary outcome measures consisted of pain intensity and muscle’s pressure pain threshold (PPT). The secondary outcome measures encompassed changes in maximal mouth opening and functional movements, as well as indicators of depression. The outcome parameters were assessed at two weeks, four weeks, six weeks and twelve weeks intervals.

#### 2.5.1 Pain

Pain was subjectively measured using the visual analog scale. Scores are recorded by making a handwritten mark on a 10-cm line representing a continuum between “no pain” and “worst pain” [13, 14]. Present pain scores were recorded for each patient.

#### 2.5.2 Pressure pain threshold

The pressure pain threshold (PPT) is the point at which a non-painful pressure stimulus transforms into a painful pressure sensation [8]. Recording the VAS score, an efficient method for pain measurement is a subjective parameter that can be assumed to be influenced by the patient’s expectation of pain relief and their ability to express the pain intensity. Therefore, the PPT was used to analyze the outcome of US therapy using an algometer. Algometry is a semiquantitative method used to locate the tender region or measure the pressure pain threshold of the involved muscle. It was measured using an algometer as pressure bearable by the patient before experiencing pain in Newtons. The measurements were performed three times with intervals of 60 seconds, and the mean value was recorded as the pressure pain threshold [5, 16].

#### 2.5.3 BDI

The Beck Depression Inventory was used to evaluate the depressive state of each patient. It consists of 21 questions. Each question has four possible answers, graded from a neutral condition (0 points) to the most severe condition (3 points). The highest possible score is 63. Zero to 13 points indicate no depression; 14 to 24 points indicate moderate depression; and scores higher than 25 points indicate severe depression [13].

#### 2.5.4 Functional movement

Mouth opening, right and left lateral movements, and protrusive movements were measured using a digital Vernier caliper.

### 2.6 Statistical analysis

The normality of the data was checked using the Shapiro-Wilk test. All the variables except mouth opening (MO) and right lateral movement (RLT) were non-normally distributed. Intergroup comparisons of age and duration of pain were performed using independent *t*-tests, and comparisons of sex and side of involvement were performed using chi-square tests. For normally distributed data, intergroup comparisons were performed using independent sample *t*-tests, and intragroup comparisons were performed using repeated-measures ANOVA and mixed model analysis. For non-normally distributed data, *i.e.*, pressure pain sensitivity (PPT), Beck Depression Inventory (BDI), left laterotrusive movement (LLT) and protrusive movement (PM), intergroup comparisons were performed using the Mann-Whitney test, and intragroup comparisons were performed using the Friedman test followed by the *post-hoc* Wilcoxon signed-rank test for paired difference tests.

An initial exploratory analysis using ANOVA was performed to assess group differences. To evaluate the effects of repeated measures of outcome parameter and individual variability, the data was subjected to mixed-effects model with outcome variables (VAS scores, PPT, MO, BDI scores) as the dependent variables and treatment groups and different time points of follow-up as fixed effects to examine their impact on the outcomes. Subjects were included as random effects to capture the repeated measures aspect. The data was found to be significantly distributed in terms of time points of outcome measurement, for VAS scores and PPT, the data followed quadratic and quartic patterns; for MO, it followed a linear pattern. A statistically significant difference was also found between the two groups regarding PPT. The data was subjected to linear mixed model analysis (LMM) for a more nuanced understanding because the ANOVA differences were statistically significant. Using mixed model analysis in addition to ANOVA produced a complementary insight since LMM is more appropriate for managing complicated data structures like random effects and repeated measurements. When the same participants are measured more than once throughout time, LMM especially manage the data. In our study, the patient outcomes were monitored at various points in time. So, LMM provided a more detailed picture of how treatment effects changed over time by more correctly accounting for within-subject variability. Missing data from follow-up or dropouts is a common problem for longitudinal studies, and this pattern was seen in our study as well where we had a drop out of 6 and 9 participants in group A and B respectively. LMM are skilled at handling all participant data points, even if some are missing at specific time points. This method, referred to as a full information maximum likelihood estimation, circumvents the biases and power loss that come with simple imputation or listwise deletion. The capacity to examine both fixed effects (like treatment groups and time points) and random effects (like individual variations in baseline attributes or treatment response) is another advantage of LMM. This dual capacity is essential to our research since it recognizes that different patients may react differently to the same treatment while also trying to determine the overall efficacy of ultrasound therapy. Data from our study has the potential to be structured on several levels (two assessors assessing results and two therapists giving treatments). LMM is more likely to accurately capture the characteristics of the data and the actual circumstances of the study.

Whilst ANOVA is effective for a more direct investigation of main effects and interactions. This combination strategy uses LMM to address the influence of nested or repeated components while facilitating an understanding of broad patterns using ANOVA. This arrangement provides for a full analysis that takes into consideration our data’s longitudinal nature as well as the layered structure of our experimental design. It is crucial to notice that non-normally distributed data should not be evaluated with LMMs. When the data satisfy the homogeneity of variances and independence of observations requirements of the assumption of normalcy, ANOVA can be used. Also, when it comes to handling situations including missing data or unbalanced designs, repeated measures, complex correlation structures, and random effects, LMMs are more adaptable than ANOVA.

The non-responders in both study groups were considered failures and were included in the final statistical analysis. *p * < 0.05 was considered as statistically significant and, *p * < 0.01 was considered as a statistically highly significant result.

## 3. Results

The study included a total of 80 participants, with a mean age of 39.08 years. 81% (65 out of 80) of the participants were females, and 18.75% (15 out of 80) were males. 85% (68 out of 80) of the participants were diagnosed with TMD with local myalgia/myofascial pain, while 15% (12 out of 80) had myofascial pain with referral. The predominant muscles involved were the masseter in 82.5% (66 out of 80) of the participants and the temporalis in 17.5% (14 out of 80). The baseline VAS score for pain was 7.62 ± 2.15 in group A and 7.22 ± 2.05 in group B. The PPT at baseline was 8.36 ± 2.48 and 9.53 ± 3.35 in groups A and B, respectively. Both study groups were similar in terms of age, sex, mean duration of pain, masticatory muscles involved and type of myalgia (*p * > 0.05) at baseline (Table 1).

**Table 1.** Comparison of the demographic characteristics of the study groups.

		Group A (n = 40)	Group B (n = 40)	*p* value
Sex				
	Female	33	32	0.778
	Male	7	8	
Age, mean ± SD, yr		37.65 ± 14.76	40.55 ± 12.69	0.340
Duration of symptoms (d)		119.93 ± 148.709	123.33 ± 163.027	0.780
Muscle involved				
	Masseter	34	32	0.559
	Temporalis	6	8	
Type of myalgia				
	Local myalgia/Myofascial pain	32	36	0.213
	Myofascial pain with referral	8	4	
Unilateral				
	Right	11	15	0.232
	Left	13	15	
Bilateral involvement		16	10	

*SD: standard deviation.*

Both groups exhibited statistically significant improvements in pain, PPT, LTT, PM and Beck Depression Scale scores, but the intergroup comparisons were not significant after therapy or at 6 or 12 weeks after treatment. There was no significant improvement in mouth opening or the RLT in either group after treatment (Tables 2,3,4).

**Table 2.** Comparison of primary outcome parameter.

Outcomes		Timeline Scores: Mean ± SD		Inter-Group Differences
		Group A (n = 40)	Group B (n = 40)	
VAS				
	Baseline	7.62 ± 2.15	7.22 ± 2.05	0.381
	2 wk	2.68 ± 2.90	3.05 ± 2.72	0.458
	4 wk	2.52 ± 3.07	2.70 ± 2.98	0.916
	6 wk	2.98 ± 2.98	2.98 ± 2.98	0.866
	3 mon	2.87 ± 3.32	2.75 ± 2.97	0.968
		*p * < 0.001	*p * < 0.001**	
PPT				
	Baseline	8.36 ± 2.48	9.53 ± 3.35	0.166
	2 wk	9.79 ± 2.96	11.58 ± 5.12	0.196
	4 wk	10.06 ± 3.04	11.98 ± 5.32	0.197
	6 wk	10.22 ± 3.10	12.34 ± 5.43	0.118
	3 mon	10.30 ± 3.08	12.37 ± 4.85	0.081
		*p * < 0.001**	*p * < 0.001**	

VAS: visual analog scale; PPT: pressure pain threshold; SD: standard deviation. **Highly statistically significant difference (p < 0.001).

**Table 3.** Comparison of secondary outcome parameters.

Outcomes		Timeline Scores: Mean ± SD		Inter-Group Differences
		Group A (n = 40)	Group B (n = 40)	
Mouth opening (MO)				
	Baseline	32.77 ± 6.42	34.70 ± 8.38	0.250
	2 wk	34.57 ± 6.25	36.77 ± 7.46	0.151
	4 wk	35.05 ± 6.25	37.58 ± 7.22	0.093
	6 wk	35.72 ± 6.46	37.92 ± 7.37	0.162
	3 mon	36.05 ± 6.66	37.90 ± 7.42	0.242
		0.173^#^	0.280^#^	
Laterotusive movement				
RLT				
	baseline	7.63 ± 1.95	7.47 ± 2.24	0.727^#^
	2 wk	7.78 ± 1.84	7.72 ± 2.03	0.886^#^
	4 wk	7.86 ± 1.78	7.81 ± 2.00	0.907^#^
	6 wk	7.88 ± 1.79	7.74 ± 2.12	0.739^#^
	3 mon	7.90 ± 1.78	7.78 ± 2.20	0.798^#^
		0.967^#^	0.957^#^	
LLT				
	baseline	8.08 ± 2.05	7.28 ± 1.71	0.090^#^
	2 wk	8.26 ± 1.96	7.50 ± 1.65	0.065^#^
	4 wk	8.34 ± 1.90	7.42 ± 1.66	0.026*
	6 wk	8.33 ± 1.91	7.42 ± 1.66	0.032*
	3 mon	8.33 ± 1.91	7.46 ± 1.58	0.038*
		*p * < 0.001**	*p * < 0.001*	
Protrusive movement (PM)				
	Baseline	4.93 ± 1.89	4.83 ± 2.14	0.992^#^
	2 wk	5.32 ± 1.78	5.15 ± 2.13	0.766^#^
	4 wk	5.40 ± 1.77	5.43 ± 2.07	0.876^#^
	6 wk	5.58 ± 1.77	5.53 ± 2.09	0.984^#^
	3 mon	5.63 ± 1.76	5.59 ± 2.14	0.984^#^
		*p * < 0.001**	*p * < 0.001**	
BDI				
	Baseline	9.02 ± 5.04	7.25 ± 3.92	0.126^#^
	2 wk	6.75 ± 5.20	5.47 ± 3.27	0.490^#^
	4 wk	6.20 ± 5.40	4.72 ± 3.32	0.374^#^
	6 wk	6.17 ± 5.68	4.40 ± 3.31	0.260^#^
	3 mon	6.47 ± 5.94	4.32 ± 3.18	0.143^#^
		*p * < 0.001**	*p * < 0.001**	
Need for alternative treatment				
	2 wk	2	3	0.476^#^
	4 wk	1	5	0.089^#^
	6 wk	1	0	0.512^#^
	3 mon	3	1	0.327^#^

*Statistically significant difference (p < 0.05), **statistically highly significant difference (p < 0.01), ^#^nonsignificant difference (p > 0.05). MO: mouth opening; RLT: right lateral; LLT: left laterotrusive movement; PM: protrusive movement; BDI: Beck Depression Inventory; SD: standard deviation.

**Table 4.** Linear mixed model analysis using outcome parameters as variables.

		*F*	*df*	*df* (res)	*p*
VAS					
	TIME	103.00	5	317.7	<0.001
	Group	3.06	1	78.0	0.996
PPT					
	TIME	21.54	5	316.3	<0.001
	Group	4.69	1	78.0	0.033
BDI					
	TIME	13.99	5	290.0	<0.001
	Group	2.77	1	79.3	0.100
MO					
	TIME	19.71	5	315.8	<0.001
	Group	2.10	1	78.0	0.151

*VAS: visual analog scale; PPT: pressure pain sensitivity; BDI: Beck Depression Inventory; MO: mouth opening.*

Six participants in group A and nine in group B did not respond to the therapy as their VAS scores did not decrease or they were not satisfied with the treatment provided. They either did not return for follow-up or were provided alternative therapy for pain relief such as splints or trigger point injections (Fig. 2). However, the baseline characteristics of these non-responders were similar to those of responders. Following the intention to treat analysis, the last recorded outcome parameters were considered as the final outcome, and the same values were considered for statistical analysis at all subsequent follow-ups.

**Fig. 2.** Flowchart detailing participants drop out of the study.

## 4. Discussion

The present study was designed to compare the therapeutic effects of two modes of US, *i.e.*, continuous and pulsed modes on TMD-M, at 3 MHz frequency.

The study included 80 participants suffering from maxillofacial region myalgia. Among the 80 patients, 15 were males (18.75%), and 65 were females (81%). This pattern of sex distribution is consistent with previous studies. Myalgia usually occurs between the ages of 30 and 60 years [10, 17, 18]. The present study revealed comparable findings, with a mean age of 39.08 years (37.65 ± 14.76 in group A, 40.55 ± 12.69 in group B). The intergroup comparison did not reveal any significant difference in age or sex distribution.

The intergroup difference in the mean duration of pain was not statistically significant. Thus, the chronicity of pain as a confounder was ruled out. In our study, the masseter was the most involved muscle in both groups (82.5%). Local myalgia and/or myofascial pain was the most common type of myalgia in both groups (85%). To prevent any bias, the patients were randomly allocated to both study groups using a simple lottery method. However, the majority of patients in both groups had local myalgia and myofascial pain, and only a few patients had myofascial pain with referral. In the intergroup comparisons, this difference was not significant. Additionally, the outcome parameters were recorded by investigators blinded to the treatment group allocation (AG and KK), thereby minimizing bias.

There was a significant reduction in the VAS score in both groups at different time intervals. This reduction in pain was highly significant from baseline to 2 weeks. However, at further follow-up visits, the pain decreased non significantly. Similar results were also reported in other studies on myofascial pain in the trapezius muscles [5, 10]. According to intergroup comparisons, the change in the VAS score was not significant at any follow-up visit. Our results are consistent with those of Yasim Fadol *et al*. [18], who compared two different duty cycles (continuous and pulse) and found no significant difference in self-reported pain between the two groups. On the other hand, Ilter L *et al*. [13] found a statistically significant improvement in resting pain scores at 6 and 12 weeks after treatment in the continuous group compared to the pulse and sham US groups. Therefore, it may be inferred that both the thermal and nonthermal effects of US waves can alleviate pain in individuals with TMD-M. A possible explanation of this study’s results can be attributed to the micro-massage being performed by the probe and the placebo effects resulting from the patient’s expectation of the treatment. The same has been supported by studies comparing therapeutic US with sham US [5, 9]. Therapeutic US has been reported to modify the electrical excitability of nerves and modify the surface morphology of cells. Altered neural membrane capacitance and reduced conductivity produce immediate analgesia in the tissue and relieve the contraction of muscle fibers [16]. This effect of therapeutic US may be responsible for immediate pain relief.

Natalia CO *et al*. [19] showed that an improvement in the PPT was not due to the application of heat alone, as there was no significant change in the pressure pain threshold in the two study groups (cryotherapy and thermotherapy). Therefore, it may be assumed that any change in PPT after US therapy may not be attributable to the thermal effect alone. Hence, PPT may be considered a better and more reliable outcome parameter than VAS for studying the effect of therapeutic US on myalgia. In our study, PPT improved significantly from baseline to 3 months in both study groups. Therefore, it may be inferred that the pressure pain threshold of the muscle increases significantly with decreasing pain. However, the intergroup comparison yielded non-significant results. This observation of our study was supported by John Z Srbely *et al*. [20] (2007) and Yasim Fadol *et al*. [18] (2016) who also reported a significant improvement in PPT after US therapy [19, 20]. lter L *et al*. [13] reported a statistically significant improvement in the degree of muscle spasm in all the groups (continuous US, pulse US, and sham). However, there was no difference in the intergroup comparisons [13]. Our findings are consistent with previously published literature that revealed a positive effect of US therapy on the PPT and no significant variation in the different modes used.

In our study, most of the patients reported a normal range of mouth opening, and only a few reported reduced mouth opening. In group A, the mean MO at baseline was 32.77 ± 6.42, which increased to 36.05 ± 6.66 at 3 months. In group B, the mean MO at baseline was 34.70 ± 8.38, which increased to 37.90 ± 7.42 at 3 months. Both intragroup and intergroup comparisons of changes in the MO were not significant. However, a study performed by Jain R *et al*. [21] (2020) reported a statistically significant improvement in the amount of mouth opening in the therapeutic US group compared to that in the conventional treatment group (medication). The results of our study contradict those of the study performed by Jain R *et al*. [21], probably because the initial MO at baseline had not significantly reduced in our study, and the sample size of the previous study was small.

In the present study, many patients reported reduced protrusive movement (PM). The values of PM increased from 4.93 ± 1.89 to 5.63 ± 1.76 in group A and from 4.83 ± 2.14 to 5.59 ± 2.14 in group B after 3 months. Both changes were statistically significant (*p * < 0.05). It was also noted that significant improvement in the PM occurred at the first and second follow-ups after the initiation of therapy in both groups. Intergroup comparisons of the mean PM from baseline to the 3-month follow-up revealed nonsignificant differences. This change in the range of the PM may be attributed to the minor role of the masseter in the protrusion [22]. The remission of pain and the masseter being the most involved muscle in the study population may have been responsible for the significant improvement in the range of protrusive movement.

The mean RLT improved non-significantly at all follow-up visits and did not differ significantly between the groups at any time interval. The mean LLT movement also increased from 8.08 ± 2.05 at baseline to 8.33 ± 1.91 at the 3-month follow-up in group A and from 7.28 ± 1.71 to 7.46 ± 1.58 at 3 months in group B; these changes were significant (*p * < 0.05) for both groups, especially from baseline to 3 months. These changes in LLT were statistically significant at the 4-week, 6-week and 3-month follow-ups when the two groups were compared. To date, no study has evaluated protrusive and excursive movements in TMD-M patients. J Nissan *et al*. [23] (2004) suggested that the right side is the preferred side for chewing by the majority of the population. This may lead to continuous loading of the masticatory muscles on the right side. In our study, there was no significant change in RLT scores, but there was a highly significant improvement in LLT scores in both groups, possibly because delayed relief was obtained on the right side compared to the left side. Although the right/left-handedness and preferred side for chewing food were not evaluated in the present study, a similar hypothesis was confirmed by J Nissan *et al*. [23, 24] (2004). Based upon the results of this study, it may be assumed that clinically, the left side muscles responded better to the treatment than did the right-side muscles at follow-up visits.

TMDs can result in pain and disability, which can have a negative impact on an individual’s daily activities, quality of life, and psychosocial functioning. The Beck Depression Inventory (BDI) is one of the most widely used self-reported measures of depression in both research and clinical practice. The Beck Depression Inventory Second Edition (BDI-II) is the most recent version of the BDI [25, 26]. Srbely JZ *et al*. [20] (2007) reported that MPS was accompanied by significant depression in most cases and that the severity of depression was related to perceived pain. Chronic pain leads to a decrease in depression and depressive symptoms with pain relief and vice versa [20]. As depression was considered a major confounder in the study plan, we evaluated patients through the BDI-II. No treatment was provided for minimal or mild depression during the treatment phase or follow-up period. Those with moderate depression were referred for expert management to the psychiatric department at the end of the study period. Another observation of the study was that the use of the BDI to evaluate depression at repeated follow-up visits is not practical because it lacks patient compliance. The patients were not willing to answer the same lengthy sets of questions at subsequent follow-up visits. Although all our study subjects responded to the questionnaire on a continuous basis, the authors felt that a shorter version of this scale would be more helpful for obtaining patient compliance.

Two studies comparing the therapeutic US with placebo showed a significant reduction in Beck Depression Scale score in the treatment group compared to the placebo group [4, 17]. Similarly, a study by Ilter L *et al*. [13] (2015) also showed no significant improvement in the BDI score in the sham ultrasound group compared to the test group. However, in both the pulse and continuous groups, the BDI improved significantly [13]. Our study also showed similar results, with a reduction in the BDI score from baseline to 2 weeks and from 2 weeks to 4 weeks in group A; similarly, a significant reduction was observed from baseline to 2 weeks, 2 weeks to 4 weeks, and 4 weeks to 6 weeks (*p * < 0.05) in group B. The intergroup comparison was not statistically significant. A reduction in BDI scores in both groups was observed along with a decrease in the VAS score for pain. At baseline, 87% of patients in group A and 92% in group B had BDI scores in the minimal range, which increased to 95% in group A and 97.5% in group B at the 3-month follow-up. However, there was no significant change in the number of patients in the mild or moderate range (Table 5). As most of the patients recruited in our study were in the minimal depression group based on the BDI score, it was not possible to determine the effect of US on the depression status of the patients.

**Table 5.** Intergroup comparison of change in percentage of patient with BDI score at baseline and 3 month follow up.

BDI score n = 80	Baseline		3-Months	
	Group A	Group B	Group A	Group B
Minimal	35 (87.5%)	37 (92.5%)	38 (95.0%)	39 (97.5%)
Mild	3 (7.5%)	3 (7.5%)	0	1 (2.5%)
Moderate	2 (5%)	0	2 (5%)	0

*BDI: Beck Depression Inventory.*

In the intention to treat patients who did not respond to therapeutic ultrasound in both groups, alternative treatments, such as muscle relaxant therapy, trigger point injection, and splint therapy, were provided, and the patients were considered to have experienced failure in the study groups. It is evident that a greater number of patients were provided alternative treatments during the initial follow-up in the pulse group than in the continuous group, but the difference between the two groups was not statistically significant. These non-responders were considered failures, and their last recorded outcome parameters were considered for statistical analysis at all subsequent follow-up visits. However, the non-responders in both groups did not differ significantly in terms of baseline parameters compared to responders.

The present study revealed good efficacy of both continuous and pulsed-mode therapeutic US for treating TMD-M, with no significant differences in the outcome parameters. Therefore, it may be assumed that the thermal and nonthermal effects of therapeutic US are effective in producing the desired pain relief, improvement in PPT, protrusive and left laterotusive movements. The improvement in the outcome parameters may be attributed to the heating effect of US waves, alteration in the nerve capacitance, and inhibition of pain transmission, thereby relaxing the contracted muscle fibers. Besides this, the micro-massage and pressure effects exerted by the US probe may also be responsible for the therapeutic effect obtained in the study participants. The result of this study were supported by previous study by Yasim Fadolet *et al*. [18] (2016), and John Z Srbely *et al*. [20] (2007) and Ilter. L *et al*. [13] (2015). Our study provides an insight into the use of a non-invasive treatment modality for the management of TMD-M. It would be unethical to not deliver any therapy to the patient in pain and make them visit for 15 days. Moreover, as no medication was prescribed during the study, it would be difficult to recall the patient in the sham US group, as no pain relief was expected in that group. So, for these ethical reasons, we did not include a sham US control group in the study. Hence, the role of compression and massage effects of the US probe itself cannot be excluded, another possible explanation for this outcome could be that the effects of the placebo on the patient were associated with the characteristics of the therapeutic environment, the excellent care provided during the study period, and the patient’s expectations of the treatment.

Strengths of the study include Randomized controlled design, use of validated outcome measures, and use of therapeutic US as first-line therapy for myalgia. Mixed model analysis was performed to analyze the data, as our study involved multiple repeated measurements taken from the same subjects over time. Mixed-effects models also addressed the individual differences in baseline characteristics or response to treatment.

Limitations of this study include lack of a sham US group, short-term follow-up, and heterogeneous sample size (including all kinds of TMD-M, *i.e.*, local myalgia and myofascial pain with or without referral); we involved more than one outcome assessor and treatment provider due to large sample size. However, care was taken to train the investigators prior to the study to administer the therapy and record the outcomes. After recalculating the sample size for LMM, it was determined to be 47 for each group, presuming a high correlation between the groups and the outcomes assessed at five different time points. This was somewhat more than the 40 in each group that we employed in our study. Therefore, further studies with diverse demographic profiles, long-term follow-up, and intra-tester and inter-tester reliability of the assessors are advised. Additionally, a comparison of different US frequencies at different modes should be performed for the treatment of TMD-M. The study’s results may prove beneficial in treating patients with TMD myalgia using non-invasive therapy.

## 5. Conclusions

Therapeutic ultrasound is a non-invasive, painless, and easy-to-use mode of therapy. This was the first double-blinded, randomized clinical study to compare the efficacy of the two modes of therapeutic US for TMD-M. A validated diagnostic criterion (DC/TMD) was used for diagnosis, and multiple outcome parameters were observed.

Both the continuous and pulse modes of US are therapeutic modalities for the management of TMD-M in terms of pain relief, protrusive and left laterotrusive functional improvement, and muscle spasm relief.

## 6. Key findings

Therapeutic US seems to be an effective therapeutic modality for the management of TMD-M in terms of pain relief.

Both modes of therapeutic ultrasound proved to be equally effective.
